# Rapid analysis of amatoxins in human urine by means of affinity column chromatography and liquid chromatography-high-resolution tandem mass spectrometry

**DOI:** 10.1038/s41598-024-72463-3

**Published:** 2024-09-13

**Authors:** Aline C. Vollmer, Claudia Fecher-Trost, Candace S. Bever, Christina C. Tam, Lea Wagmann, Markus R. Meyer

**Affiliations:** 1 Department of Experimental and Clinical Toxicology, Institute of Experimental and Clinical Pharmacology and Toxicology, Center for Molecular Signaling (PZMS), Saarland University, Kirrberger Str., Building 46, 66421 Homburg, Germany; 2 Foodborne Toxin Detection and Prevention Research Unit, Western Regional Research Center, Agricultural Research Service, United States Department of Agriculture, Albany, CA USA

**Keywords:** Method development, Amatoxins, Monoclonal antibody, Magnetic beads, Affinity column chromatography, Liquid chromatography-high-resolution mass spectrometry, Laboratory techniques and procedures, Bioanalytical chemistry, Mass spectrometry

## Abstract

Analysis of amatoxins is of great importance as these cyclic peptides contribute to a high number of fatalities each year. Development of analytical approaches needs to focus on rapid, sensitive, and reliable methods. By establishing an affinity column chromatography-based assay using the monoclonal amanitin antibody AMA9G3 and liquid chromatography (LC) coupled to high-resolution mass spectrometry (HRMS) for the trace detection of α-, β-, and γ-amanitin in human urine samples to confirm ingestion, we report the first approach that extents the current status of amatoxin analysis. The presented procedure allows detection of amatoxins in human urine down to 1 ng/mL. The method was successfully validated qualitatively for α- and γ-amanitin according to international recommendations. A proof of concept was performed by analyzing 37 urine samples after suspected amatoxin consumption submitted for regular clinical toxicological analysis. Using this antibody-based enrichment strategy, acute amatoxin intoxications can be determined within 90 min and due to the high sensitivity and selectivity, a comparable approach using target specific antibodies may also be used for other toxicological relevant peptides.

## Introduction

Globally, approximately 5000 kinds of fungi have been discovered and about 150 in Europe are known to be poisonous^1^. Genera such as *Amanita, Lepiota,* and *Galerina* contain amatoxins, which contribute to high rates of fatalities and *A. phalloides* (death cap) is responsible for 90–95% of human deaths after amatoxin ingestion^2^. Consumption leads to irreversible structural cell changes and necrotic damage of organs such as liver and kidney^3^. Thus, immediate therapeutic intervention is needed and requires the development and application of reliable and fast analytical methods to identify amatoxins in biological samples^3^.

Multiple different approaches for the detection of amatoxins have been developed such as radioimmunoassay (RIA), enzyme-linked immunosorbent assay (ELISA), lateral flow immunoassay (LFIA), and liquid chromatography-high-resolution tandem mass spectrometry (LC-HRMS/MS)^4–7^. Detection of amatoxins in urine is possible with a commercially available RIA. However, the tracer shows limited stability (two months or less) and the assay is only available during mushroom season thus hindering detection of suspected amatoxin poisonings due to ingestion of e.g., deep frozen amanita mushrooms throughout the year^2^. A commercial ELISA kit available throughout the year detects α- and γ-amanitin until 0.2 ng/mL and indicates higher selectivity than the RIA^2,4^. Both the RIA and ELISA do not rely on expensive laboratory equipment but are time-consuming and require specialized reagents^2^. Recently, Bever et al. developed an LFIA for mushroom toxins which allows detection of amatoxins as little as 10 ng/mL in urine. This LFIA offers the opportunity for simple and rapid point-of-care testing without the need of trained personnel. However, LFIA findings always require confirmation by an additional analytical method such as LC-HRMS/MS as it is prone to false-positive or false-negative results due to possible cross-reactivities e.g., with phallotoxins but also with other compounds contained in *Amanita* species^2,8^.

Within the last decade, LC-HRMS/MS has been commonly used for detection of amatoxins in various matrices^5,6,9^. Whereas LC offers selectivity and separation efficiency, MS provides structural information, unique compound identification by mass, and increased detectability^2^. Nevertheless, sample extraction and sophisticated equipment is requisite^10^. Hence, combining antibody enrichment and LC-HRMS/MS for analysis of the target compounds would provide high level of sensitivity, specificity, and selectivity^11^.

Urine is used as the matrix for detection since amatoxin symptoms often present approximately 12 h after ingestion and amatoxins may be already eliminated from plasma and concentrated in urine compared to other body fluids^4,12^. Due to potential interfering compounds in urine, solid-phase extraction (SPE)-based methods were commonly developed^2,5^. However, amatoxin extraction via SPE often requires larger volumes of different (organic) solvents and involves multiple time-consuming working steps including sample pretreatment, preconditioning of the SPE cartridge, sample loading, washing steps, elution of the target compounds, evaporation, and finally reconstitution^6^. Therefore, antibody-based enrichment strategies for proteins and peptides using magnetic separation may be a more efficient alternative to conventional SPEs. Antibodies can selectively and strongly bind to their target analyte, allowing the remaining sample matrix to be removed. When target analytes are eluted from the antibody affinity column, the sample typically has less interfering substances, making it very amenable to detection via e.g., MS. Particularly, monoclonal antibodies (mAb) are produced to target one specific epitope on an antigen, making them useful in such antibody-based procedures^13^. Furthermore, an appropriate amount of antibody for the assay needs to be determined to extract the target compounds reliable and reproducible.

In this study, we developed an analytical procedure to detect α-, β-, and γ-amanitin in human urine using magnetic bead-based affinity column chromatography followed by LC-HRMS/MS analysis. This new approach was used to detect these toxins in human urine samples from suspected amatoxin poisonings as a proof of concept and the results were qualitatively validated according to international recommendations and compared to conventional SPE-based procedures^5,6^.

## Methods

### Chemicals

Alpha (α)- and beta (β)-amanitin were purchased from VWR International GmbH (Darmstadt, Germany), gamma (γ)-amanitin from Abcam (Berlin, Germany), methyl-γ-amanitin was donated by Prof. Dr. Heinz Faulstich (Max-Planck-Institute for Cell Biology, Ladenburg, Germany). Econo-Chromatography Columns (1.0 × 10 cm) were obtained from Bio-Rad Laboratories (Feldkirchen, Germany), Dynabeads Protein A and G, Pierce Crosslink Magnetic IP/Co-IP Kit, Pierce Protein A/G agarose, PageRuler Plus Prestained Protein Ladder, DynaMag-2, Bolt 4–12% BT gradient gels, and Vivaspin 20 centrifugal concentrator (MWCO 100,000 Da) from Fisher Scientific GmbH (Schwerte, Germany). Methanol, acetonitrile (ACN), and sodium chloride (NaCl) were bought from VWR International GmbH (Darmstadt, Germany), 2-mercaptoethanol (≥ 99%), glycerol (≥ 99.5%), glycine (≥ 99%), sodium hydrogen carbonate (NaHCO_3_, ≥ 99%), and tris-(hydroxymethyl)-aminomethan (TRIS) Pufferan® (≥ 99.9%) from Carl Roth GmbH and Co. KG (Karlsruhe, Germany), sodium azide (NaN_3_) from AppliChem GmbH (Darmstadt, Germany), polypropylene tubes from Greiner Bio-One GmbH (Frickenhausen, Germany), protein LoBind tubes and silanized vials from Eppendorf (Hamburg, Germany). Water was purified with a Millipore filtration unit (18.2 Ω × cm water resistance) from Merck (Darmstadt, Germany).

### Antibody purification

Mouse ascites fluid containing mAb (AMA9G3) was kindly provided by the United States Department of Agriculture via a material transfer agreement (Albany, USA). AMA9G3 was purified before sample preparation. First, 2 mL of Pierce Protein A/G agarose were resuspended for 2 min (Vortex Genie 1 TouchMixer) and transferred to an Econo-Chromatography Column followed by three washing steps using 5 mL of 1 × TRIS-buffered saline (TBS, pH 7.4) each. A volume of 3 mL ascites fluid was diluted using 22 mL of 1 × TBS in a polypropylene tube and then applied onto the column overnight at 4 °C using a peristaltic pump (Pharmacia Biotech). Afterwards, the protein A/G agarose was washed with 1 × TBS until the absorption A280 nm in the washing solution reached zero. Then, one further washing step using 25 mL of 1 × TBS was conducted. AMA9G3 was eluted using 2.5 mL of aqueous glycine buffer (100 mM, pH 2.7) into 1 mL of neutralization buffer (aqueous 0.1 M NaHCO_3_ containing 0.1 M NaCl, pH 8.5). Finally, a pH value of 7.5 should be obtained which has to be tested prior to antibody elution. Absorption was measured from each eluate (1–5) using the NanoPhotometer N60. Eluates 1–3 were combined and concentrated using a Vivaspin 20 centrifugal concentrator in a vacuum centrifuge (Sigma 3-16PK). After measuring the final concentration, purified AMA9G3 was diluted using neutralization buffer and stored at 4 °C. To keep and reuse the protein A/G column, a mixture of 10 mL of 1 × TBS and 62.5 µL NaN_3_ (8%) was prepared, applied, and the column stored at 4 °C.

### Gel electrophoresis

To analyze antibody purity, sodium dodecyl sulfate–polyacrylamide gel electrophoresis (SDS-PAGE) was performed at room temperature (RT) using Bolt 4–12% gradient gels. Purified AMA9G3 (40 µg) was mixed with 20 µL sample buffer (120 mM TRIS HCl, pH 6.8, 8% SDS, 20% glycerol, and 0.72 M 2-mercaptoethanol) in a 1.5 mL protein LoBind tube. PageRuler Plus Prestained Protein Ladder was used as protein marker. Prepared antibody solution was denatured for 20 min at 60 °C (ThermoMixer HTMR-133) before loading into minigels. The SDS-PAGE gels were run for 45 min starting with 80 V and increasing to 200 V after 15 min. Afterwards, the gel was visualized with 0.125% (*w/v*) Coomassie Brilliant Blue R250 overnight.

### Antibody affinity column preparation

Binding of AMA9G3 to protein A and G magnetic beads used as affinity ligands was performed according to a published procedure with some modifications^14^. Dynabeads Protein A and G were resuspended by shaking for 1 min (VWR VV3, level 2.5) and 25 µL of each magnetic bead suspension was transferred to a 1.5 mL protein LoBind tube and magnetized for 2 min using the DynaMag-2. The supernatant was removed and two washing steps were conducted using 1 mL of 1 × TBS each, shaking for 10 s, magnetizing for 2 min, and pipetting off the supernatant. Washed Dynabeads Protein A and G were incubated with 100 µL antibody working solution (see stock and working solutions) for 1 h at 4 °C under gentle shaking (HLC Thermomixer KTMR-133) followed by another washing step as mentioned above.

### Stock and working solutions

Stock solutions of α-, β-, and γ-amanitin (1 mg/mL) as well as methyl-γ-amanitin (internal standard, IS, 2.5 mg/L) were prepared separately in methanol and handled in amber-colored glass vials. Working solution containing α-, β-, and γ-amanitin was prepared in purified water and freshly spiked into blank urine prior to affinity column chromatography. All solutions were stored at − 20 °C. AMA9G3 was purified before use, diluted using neutralization buffer to a final concentration of 1 mg/mL and stored at 4 °C for at least two months. For antibody immobilization, a working solution containing 20 µL of AMA9G3 (20 µg/100 µL), 5 µL IP Lysis/Wash Buffer, 5 µL 20 × coupling buffer (composition of buffers, see Pierce Crosslink Magnetic IP/Co-IP Kit), and 70 µL purified water were used.

### Affinity column chromatography

Antibody binding was followed by extraction of the target compounds. One ml of urine was added to magnetic beads immobilized with AMA9G3 followed by 5 s of shaking (VWR VV3, level 2.5) and incubation for 1 h at 22 °C under gentle shaking (800 rpm, CellMedia ThermoMixer TS pro). Afterwards, urine was discarded and four washing steps following the same procedure as mentioned above (see antibody affinity column preparation) were included. Elution was performed by incubating the bound amatoxins in 150 µL of aqueous glycine buffer for 5 min at 22 °C under gentle shaking which was repeated three times. 400 µL of the extract were transferred to a fresh 1.5 mL protein LoBind tube and methyl-γ-amanitin as IS was added (20 ng/mL final concentration). For injection, 50 µL were transferred into a silanized vial followed by the injection of 10 µL onto the LC-HRMS/MS system.

### LC-HRMS/MS conditions

Urine samples were analyzed using a Thermo Fisher (TF) Vanquish Duo ultra-high performance LC system consisting of a degasser, a binary pump, and a dual split sampler HT (Thermo Fisher Scientific, TF, Dreieich, Germany) coupled to a TF Orbitrap Exploris 120 system equipped with a heated electrospray ionization (HESI)-II-source. The instrument was calibrated prior to analysis according to the manufacturer´s recommendations using external mass calibration. Gradient elution was performed on a TF Accucore PhenylHexyl column (100 mm × 2.1 mm, 2.6 µm particle size) using eluent A (water with ammonium acetate (10 mM, pH 5)) and eluent B (ACN:methanol (1:1, *v/v*) with water (1%, *v/v*), and formic acid (0.1%, *v/v*)). For chromatographic separation of the amatoxins, the gradient was programmed as follows: 0–6.5 min hold 1% B, 6.5–9.5 min up to 50% B, 9.5–9.51 min up to 99% B, 9.51–14.5 min hold to 99% B, and 14.51–15 min down to 1% B. The flow rate was set to 300 μL/min from 0 to 14.5 min and decreased to 50 μL/min until 15 min. HESI-II-source conditions were as follows: sheath gas, 60 arbitrary units (AU); auxiliary gas, 10 AU; spray voltage, 3.00 kV (negative polarity); vaporizer temperature, 320 °C; ion transfer capillary temperature, 320 °C; and S-lens radio frequency level, 60.0. General parameters were: polarity, negative; default charge, 1. Parameters for receiving full scan (FS) data in negative mode were as follows: resolution, 60,000; normalized automatic gain control (AGC) target, standard; maximum injection time (IT), auto; and scan range, mass-to-charge ratio (*m/z*) 850–950. Settings for data dependent MS^2^ (ddMS^2^) were as follows: resolution, 30,000; higher energy collision dissociation, 40 V; normalized AGC target, 100%; maximum IT, 250 ms; intensity threshold, 1.0 e^2^; isolation window, *m/z*, 2.

### Method validation

Method validation for qualitative analysis was performed for α- and γ-amanitin using 10 ng/mL spiked urine samples (validation samples) according to international recommendations^15–17^. Selectivity, carry-over, and different stability data were evaluated in accordance with the “ICH guideline M10 on bioanalytical method validation and study sample analysis”, matrix effects (ME) and recoveries (RE) in accordance with Matuszewski et al.^17,18^. Drugs detected in human urine samples used for the selectivity study were listed in the Supplementary material Table S1. Limits of identification (LOI) and detection (LOD) were additionally determined^15,16^. Details on experiments as well as acceptance criteria and used software for data handling are given in the Supplementary material.

### Proof of concept

A total of 37 urine samples from suspected amatoxin intoxications submitted to the authors laboratory for regular clinical toxicological analysis were analyzed and results compared to SPE-based procedures^5,6^. All methods were carried out in accordance with relevant guidelines and regulations.

## Results

### Development of an antibody-based enrichment assay

Following AMA9G3 purification, heavy and light chains of approximately 50 kDa as well as 25 kDa were visualized as shown in Fig. S1. AMA9G3 was successfully bound non-covalently to magnetic beads surface coupled with Protein A and G. Other commercially available mAb were not tested. This antibody affinity column significantly enriched amatoxins from at least 1 ml of urine. Reproducibility of recovery indicated by coefficients of variation (CV) after affinity column chromatography was ≤ 15% for α- and γ-amanitin and at 22% for β-amanitin (see Table 1).

**Table 1:** Main characteristics of the amatoxins and internal standard (IS) including *m/z* of the precursor [M–H]^−^ and qualifier ion, retention time (t_R_), limit of identification (LOI), limit of detection (LOD), and reproducibility of the antibody binding followed by affinity column chromatography (*n* = 3). *m/z*, mass-to-charge ratio; n.d., not determined; CV, coefficient of variation.

Analyte	Precursor ion, *m/z*	Qualifier ion, *m/z*	t_R_ (min)	LOI (ng/mL)	LOD (ng/mL)	CV (%)
α-Amanitin	917.3458	205.0441	9.5	1	1	± 15
β-Amanitin	918.3298	205.0441	9.3	1	1	± 22
γ-Amanitin	901.3514	205.0441	9.8	1	1	± 4.5
Methyl-γ-amanitin (IS)	915.3665	915.3665	10.3	n.d	n.d	n.d

### Analysis of amatoxins using LC-HRMS/MS

**Fig. 1:**
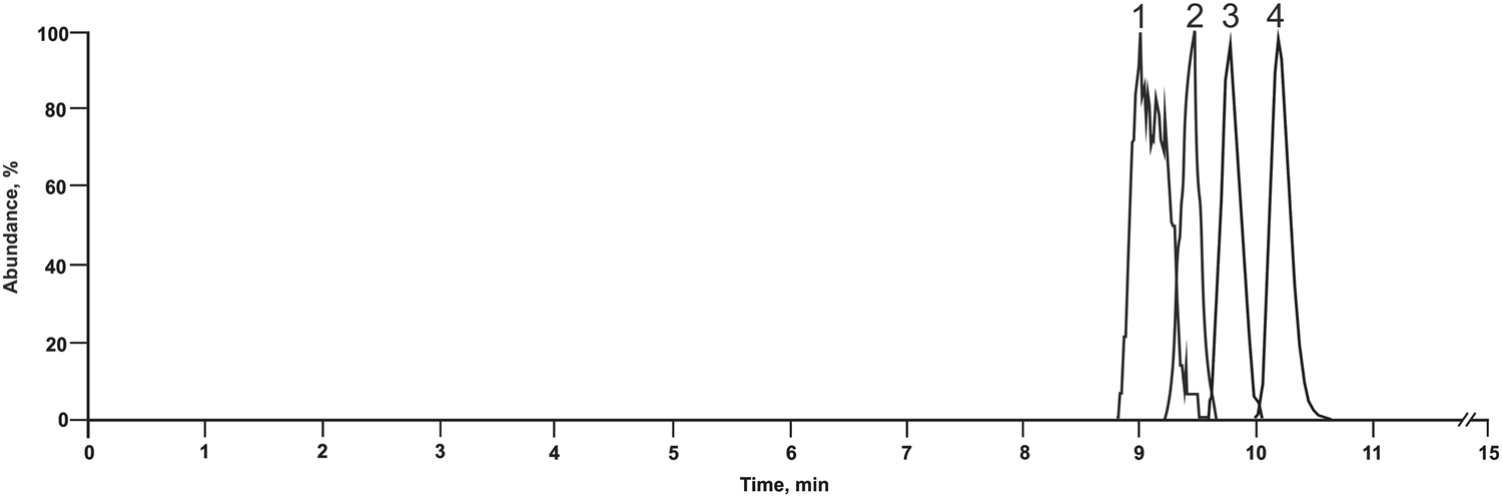
Reconstructed ion chromatograms (*m/z*) of α-, β-, and γ-amanitin and the internal standard (IS) spiked into blank urine and extracted using 50 µg/100 µL AMA9G3. All peaks are at 100% abundance. 1: β-amanitin (1 ng/mL); 2: α-amanitin (1 ng/mL); 3: γ-amanitin (1 ng/mL); 4: methyl-γ-amanitin (IS, 20 ng/mL).

Identification of the amatoxins was possible using their deprotonated (mass-to-charge ratio *m/z* of [M–H]^−^) precursor ion in FS mode, the specific MS^2^ fragment, and retention time. Methyl-γ-amanitin was suitable as IS and could be used for performance monitoring of the LC-HRMS/MS procedure but may also be replaced by other suitable peptides. Chromatographic separation was achieved within 15 min total runtime. Determined analytical data of the amatoxins and the IS including their LOI and LOD are summarized in Table 1. Reconstructed ion chromatograms of the *m/z* of the amatoxins and the IS are depicted in Fig. 1. The fragmentation patterns of the amatoxins are shown in Fig. S3.

### Method validation

Selectivity was shown to be given as no interferences were present at the respective retention time. Reconstructed ion chromatograms obtained after analysis of a urine sample used for the selectivity study are depicted in Fig. S2. Carry-over was not observed after injection of a urine sample spiked with 50 ng/mL α- and γ-amanitin. ME and RE are given in Table 2. ME for α- and γ-amanitin were determined to be 105% and the RE for both compounds were determined to be 19%. Stock and working solutions of α- and γ-amanitin were stable for 6 weeks stored at − 20 °C since no degradation over 15% could be observed. Spiked urine samples were stable for 24 h stored in the refrigerator (4 °C) and at RT (22°). Analytes in matrix were also not affected by degradation if stored over four weeks at − 20 °C. Freezing for 24 h followed by thawing to RT and further affinity column chromatography also indicated no degradation of α- and γ-amanitin. Results of the stability testing are summarized in Table 3.

**Table 2:** Matrix effects (ME), recoveries (RE), and CVs of α- and γ-amanitin (*n* = 6, 10 ng/mL). CV, coefficient of variation.

Analyte	Validation sample (CV, %)	
	ME (%)	RE (%)
α-Amanitin	105 (4.2)	19 (14)
γ-Amanitin	105 (4.4)	19 (13)

**Table 3:** Results of the stability testing for α- and γ-amanitin, and internal standard (IS). Spiked blank urine samples stored under different conditions and extracts stored in the autosampler (*n* = 3, 10 ng/mL), peak area deviations of measurement at timepoint t_0_ compared to t_1_, % and CVs, %. CV, coefficient of variation; n.d., not determined.

Analyte	Validation sample, (CV, %)					
	Short term (24 h, 4 °C)	Benchtop (24 h, 22 °C)	Long term (4 weeks, 20 °C)	Freeze/thaw (24 h, − 20 °C)	Autosampler (1 day, 20 °C)	Autosampler (4 days, 20 °C)
α-Amanitin	100 (8.9)	93 (13)	110 (3.8)	110 (7.7)	94 (2.5)	86 (2.6)
γ-Amanitin	101 (6.0)	93 (15)	110 (5.0)	109 (8.6)	94 (2.1)	86 (1.6)
Methyl-γ-amanitin (IS)	n.d	n.d	n.d	n.d	95 (1.3)	88 (1.7)

### Proof of concept

Table 4 summarizes the results from the proof of concept study. Results could be compared to those after SPE extraction^5,6^. For samples indicated as “positive” for amatoxins, the presence of α-, β-, and/or γ-amanitin was confirmed. Using affinity column chromatography, in six samples (sample ID 8, 12, 27, and 31–33) α- and γ-amanitin could be detected and in two cases (sample ID 26 and 30), only α-amanitin. Further analyzed 29 urine samples (sample ID 1–7, 9–11, 13–25, 28–29, and 34–37) were negative.

**Table 4:** Detection results of the 37 suspected amatoxin poisonings in urine submitted for toxicological analysis including sample ID, sampling time after assumed ingestion, results of the affinity column chromatography and former developed methods^5,6^; h, hours; +, positive for α-, β-, and/or γ-amanitin; −, negative for α,-β-, and/or γ-amanitin; n.g., not given.

Sample ID	Sampling time after assumed ingestion (h)	Detection result using immunoprecipitation	Detection result using solid-phase extraction^5,6^
1	n.g	−	−
2	18	−	−
3	n.g	−	−
4	n.g	−	−
5	30	−	−
6	28	−	−
7	n.g	−	−
8	n.g	+	+
9	11	−	−
10	n.g	−	−
11	n.g	−	−
12	n.g	+	+
13	144	−	−
14	n.g	−	−
15	192	−	−
16	n.g	−	−
17	n.g	−	−
18	43	−	−
19	> 14	−	−
20	n.g	−	−
21	n.g	−	−
22	25	−	−
23	25	−	−
24	n.g	−	−
25	n.g	−	−
26	49	+	+
27	n.g	+	+
28	n.g	−	−
29	26	−	−
30	n.g	+	+
31	n.g	+	+
32	n.g	+	+
33	14	+	+
34	59	−	−
35	6	−	−
36	n.g	−	−
37	n.g	−	−

## Discussion

Several analytical techniques and sample preparation procedures exist for the detection and/or quantification of amatoxins in different biosamples^2,5,7,9,19,20^. As rapid and accurate identification of amatoxin poisonings is crucial, this study aimed to develop an antibody-based enrichment strategy followed by LC-HRMS/MS analysis to detect α-, β-, and γ-amanitin in human urine.

Detection and identification of the amatoxins was possible until at least 1 ng/mL in urine, which is comparable to previous reports even though different sample preparation techniques had to be performed^6,19,21^. The criterion for the determination of the LOD was the presence of the FS peak of the compounds at the respective retention time. The determination of the LOI was based on the detection of the specific MS^2^ fragment in addition to the FS peak. As shown in Fig. S3, the fragment ion at *m/z* 205.0441 can be used as qualifier ion based on its presence in each of the MS^2^ spectra. It should further be considered that α-, β-, and γ-amanitin have different precursor ions. Helfer et al. also described the fragment ion at *m/z* 257.1142, which was only detected in the MS^2^ spectrum of α-amanitin^19^. Since the validation sample, which contains known amounts of the amatoxins, was always analyzed as reference in addition to the patient sample, results can be compared. Moreover, comparison of the current procedure to methods by Maurer et al. and Bambauer et al. also reveals further benefits^4,6^. After immunoaffinity extraction performed by Maurer et al., LODs for α- and β-amanitin were 2.5 ng/mL. Here, we could detect α-, β-, and γ-amanitin down to 1 ng/mL. The sample volume could be reduced down to 1 mL instead of 1.46 mL or even 5 mL. In addition, Bambauer et al. did not use an automated SPE approach but all extractions containing several time-consuming working steps were performed manually. The time to identify an amatoxin intoxication is more than three hours, whereas with this antibody-based approach, results are available in less than 90 min. Antibodies tend to form insoluble aggregates in organic solvents such as methanol, trifluoroethanol, or dimethylsulfoxide, which can also lead to decreased antibody binding capacity depending on the antibody used^22^. Therefore, compared to SPE-based methods, the extraction of the target compounds does not rely on organic solvents but only on aqueous buffers^5,6^. In general, amatoxins can be analyzed in negative or positive ionization mode^9,19^. As reported in literature, positive ionization can yield higher ME owing to *N*-acyl amino acid-derived compounds co-eluting with the target analytes^19^. Nevertheless, measuring in positive mode was tested during method development with the result that the amatoxins were not detectable at 1 ng/mL in urine. Therefore, analysis was performed in negative ionization mode, which is less susceptible to ME.

Magnetic beads are available with various surface chemistries. Selection criteria for using Dynabeads Protein A and G for the antibody-based enrichment of the amatoxins were high coupling efficiency, low background binding of other proteins or peptides, the possibility to develop a time-saving affinity column chromatography procedure, and the use of LC-HRMS/MS analysis as downstream application. As this study examined non-covalent attachment of the antibody to magnetic beads, the final extract contained the amatoxins next to AMA9G3 after elution. For future studies, covalent binding approaches would be worth exploring^4^. Consequently, the sensitivity (LOD) as well as the RE may benefit from a defined orientation of the antibody coupled to the solid support^23^. Nevertheless, for efficient usage of purified and non-covalent attached AMA9G3 and given the fact that α-amanitin is predominantly found in mushrooms and β-amanitin indicates lower binding affinity to AMA9G3, method validation was only performed for α- and γ-amanitin^7,24^. As first symptoms of amatoxin poisonings typically occur after a latency period of 8–10 h, sufficient sensitivity is required as hospitalization and thus urine sampling tend to be performed late^3^. Therefore, different volumes of magnetic beads (100 µL, 50 µL, and 25 µL of each magnetic bead suspension) as well as different amounts of AMA9G3 (10–80 µg/100 µL) were evaluated experimentally with regards to the detection of α-, β-, and γ-amanitin until 1 ng/mL in urine. However, using higher amounts of magnetic beads (more than 25 µL) or AMA9G3 (more than 50 µg/100 µL) did not lower the LOD.

Apart from analytical sensitivity, investigation of ME is equally important as matrix compounds might co-elute with the target analytes and thus leading to ion suppression or enhancement^25^. Antibody-based enrichment allows for selective isolation and thereby lowering potential ME. This was also shown in this study for α- and γ-amanitin as presented in Table 2 where ME were determined to be 105%, which suggests minimal interferences. In comparison, previous work published by Abbott et al. using a SPE-based sample preparation for extraction of amatoxins, reported ME ranged from 21 to 41%^24^. RE were only determined to be 19% although it was found to be reproducible with CVs within 15% and as already mentioned, clinically relevant amatoxin concentrations in urine can still be detected.

Samples analyzed in the context of clinical or forensic toxicology often contain many different drugs including their metabolites but also toxins or endogenous compounds. Therefore, impact of interferences usually contained in such samples need to be checked^16^. Selectivity was tested using human urine samples containing various drugs but no amatoxins. Nevertheless, it should be considered that the influence of further metabolites or endogenous matrix compounds cannot be excluded. During method validation, evaluation of stability was performed to ensure that amatoxins undergo no degradation in matrix under different storage conditions. Although amatoxin poisonings need to be determined urgently, long term stability might also be important if e.g., re-analysis of samples must be performed or in the context of forensic toxicology. Certainly, storage temperatures for the evaluation of long term stability should be in line with method purpose^15^. If samples are usually stored at − 20 °C, the long term stability study should also be conducted at − 20 °C which was the case referring to this study.

Thirty-seven urine samples submitted for toxicological analysis were analyzed for detection of amatoxins and results compared to former developed SPE-based procedures. Overall, results obtained were in line with reference methods based on SPE^5,6^. Amatoxins are mainly excreted into urine during the first 72 h after ingestion but can still be present after a few days^12^. Unfortunately, information regarding exposure to amatoxin-containing mushrooms or the time between consumption and urine sampling is often unknown. A large time gap between sampling and assumed ingestion as well as the fact that most mushrooms contain much less γ-amanitin compared to α-amanitin might explain that only α-amanitin but not γ-amanitin could be detected after analyzing sample IDs 26 and 30^24^.

Diagnostic determinations about an amatoxin poisoning must be determined early using fast, sensitive, and reliable analytical methods. This approach certainly has its limitations as some preparatory steps must be carried out before analysis. However, the presented procedure offers several benefits such as better time-efficiency compared to manually performed SPEs, reduction of organic solvents during the extraction, and less ME. AMA9G3 can be attached to protein A and G magnetic beads and stored at 4 °C as this antibody has shown to be stable for at least two months during method development and validation. This is also the period in which this antibody-bead column can be used for extraction of the amatoxins. Once an amatoxin intoxication must be diagnosed, the main steps to be performed consist of the affinity column chromatography followed by LC-HRMS/MS analysis making this strategy a valuable tool in clinical toxicology. This approach also offers potential for further investigations. In literature, studies regarding the metabolism of α-amanitin report the in vitro formation of one glucuronide metabolite in trace amounts^26,27^. Therefore, a question worth investigating is whether amatoxins are metabolized in humans and whether the monoclonal antibody AMA9G3 could also be used to detect metabolites if they are formed.

## Conclusions

Here, we report the first study using magnetic bead-based affinity column chromatography for the detection of α-, β-, and γ-amanitin in human urine followed by LC-HRMS/MS analysis. Amatoxin identification was possible at least down to 1 ng/mL in urine, which is sufficient and similar to previous methods. Although AMA9G3 has to be purified and affinity columns have to be prepared as preliminary work, this presented approach allows determination of an acute amatoxin intoxication in less than 90 min and for reduced ME as the sample matrix is highly cleaned up during the extraction. The strategy was successfully applied to detect amatoxins in urine samples after suspected consumption of mushrooms and results obtained were in line with reference methods based on SPE. The current method demonstrated the potential to identify intoxications with amatoxins providing essential information to support diagnosis and further therapy in hospital. Furthermore, as this workflow can be adopted easily, other toxicological relevant peptides may be extracted using target specific antibodies.

## Supplementary Information

Supplementary Information.

## Data Availability

Data will be made available on request via email: m.r.meyer@mx.uni-saarland.de
