# Supplementary material for: Rapid analysis of amatoxins in human urine by means of affinity column chromatography and liquid chromatography-high-resolution tandem mass spectrometry
Source: Sci Rep. 2024 Sep 13;14:21397. doi: 10.1038/s41598-024-72463-3 (PMC11399331; doi:10.1038/s41598-024-72463-3)
Supplement: Supplementary file 1 — Supplementary Information. [file 41598_2024_72463_MOESM1_ESM.pdf]

## Supplementary material

# Rapid Analysis of Amatoxins in Human Urine by Means of Affinity Column Chromatography and Liquid Chromatography-High-Resolution Tandem Mass Spectrometry

Aline C. Vollmer[a], Claudia Fecher-Trost[a], Candace S. Bever[b], Christina C. Tam[b], Lea Wagmann[a], Markus R. Meyer[a]

[a]Department of Experimental and Clinical Toxicology, Institute of Experimental and Clinical Pharmacology and Toxicology, Center for Molecular Signaling (PZMS), Saarland University, Homburg, Germany

[b]Foodborne Toxin Detection and Prevention Research Unit, Western Regional Research Center, Agricultural Research Service, United States Department of Agriculture, Albany, CA, USA

**Data handling and method validation**

Statistical analysis was performed using Microsoft Excel 2010 (Redmond, WA, USA). ACD/Chem Sketch freeware version 2015 was used for the calculation of the exact masses of the amatoxins as well as the internal standard (IS) and TF Xcalibur Qual Browser version 4.1 for qualitative data evaluation. Qual Browser settings were as follows: detector type, MS; peak algorithm, genesis; plot type, mass range; *m/z* of each substance; mass tolerance, 5 ppm; mass precision, 4 decimals; smoothing, not enabled. Extracted ion chromatograms of the analytes were edited using CorelDraw X7 Version 17.0.0.491 (Munich, Germany). Method validation for α- and γ-amanitin covering selected parameters was performed according to international recommendations [1-3]. Selectivity testing was performed using urine samples from six different donors which were extracted, analyzed, and evaluated for interferences. For carry-over testing, eight extracted blank urine samples were injected after a urine sample spiked with 50 ng/mL of α- and γ-amanitin. Matrix effects (ME) and recoveries (RE) were determined according to Matuszewski et al. using three different sample sets ($n = 6$, concentration of α- and γ-amanitin, 10 ng/mL) [4]. The first sample set (neat standard) was prepared in aqueous glycine buffer (100 mM, pH 1.8). Blank urine was spiked with α- and γ-amanitin before affinity column chromatography (sample set 3) or after affinity column chromatography (sample set 2). For α- and γ-amanitin, ME were calculated using the ratio of the peak area in the presence of matrix (sample set 2) and the peak area in absence of matrix (sample set 1). Division of mean peak areas of sample set 2 by those of sample set 1 were used for calculation of the ME. Coefficients of variation (CV) for α- and γ-amanitin should not be greater than 15%. Division of mean peak areas of sample set 3 by those of sample set 2 was used for calculation of the RE. Stability of the stock and working solutions of α- and γ-amanitin as well as the IS was tested over a period of six weeks in purified water ($n = 3$, concentration of α- and γ-amanitin and IS, 500 ng/mL). CVs not greater than ±15% were defined to be acceptable. Autosampler stability of processed samples was investigated over a period of four days (extracts stored at 20 °C) next to short-term

stability (spiked urine samples stored 24 h at 4 °C), benchtop stability (spiked urine samples stored 24 h at 22 °C), and long-term stability (spiked urine samples stored four weeks at -20 °C). One freeze and thaw cycle was conducted for the freeze and thaw stability (spiked urine samples stored for 24 h at -20 °C). Urine samples spiked with α- and γ-amanitin (final plasma concentration, 10 ng/mL, $n = 3$) were extracted and analyzed immediately after preparation ($t_0$) and after the appropriate storage condition ($t_1$). Peak area deviations as well as CVs not greater than ±15% were defined to be acceptable.

## References


1. Wille, S.M.R., et al., *Update of Standard Practices for New Method Validation in Forensic Toxicology.* Curr Pharm Des, 2017. **23**(36): p. 5442-5454.
2. Peters, F.T., O.H. Drummer, and F. Musshoff, *Validation of new methods.* Forensic Sci Int, 2007. **165**(2-3): p. 216-24.
3. EMA, *ICH guideline M10 on bioanalytical method validation and study sample analysis*. 2022, European Medicines Agency: Amsterdam, The Netherlands.
4. Matuszewski, B.K., M.L. Constanzer, and C.M. Chavez-Eng, *Strategies for the assessment of matrix effect in quantitative bioanalytical methods based on HPLC-MS/MS.* Anal Chem, 2003. **75**(13): p. 3019-30.
5. Bever, C.S., et al., *Lateral flow immunoassay (LFIA) for the detection of lethal amatoxins from mushrooms.* PLoS One, 2020. **15**(4): p. e0231781.

**Table S1** Additional drugs included in the selectivity study.

| | |
|---|---|
| Amphetamine | Metformin |
| Amlodipine | Midazolam |
| Aripiprazole | Promethazine |
| Clozapine | Quetiapine |
| Furosemide | 9-Hydroxy-risperidone |
| Lamotrigine | Sitagliptin |
| Lidocaine | Tetrahydrocannabinol-carboxylic acid |
| Metamizole | Zolpidem |

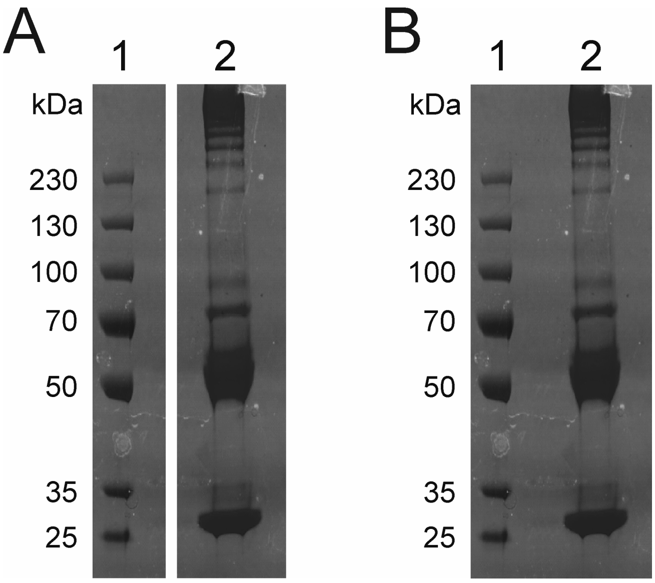


**Fig. S1** Gel electrophoresis of purified AMA9G3 which was fully characterized by Bever et al. [5] (1 PageRuler Plus Prestained Protein Ladder (protein marker), 2 AMA9G3 (40 µg) in sample buffer). (A) Cropped gel with grey background. (B) Full-length gel with grey background.

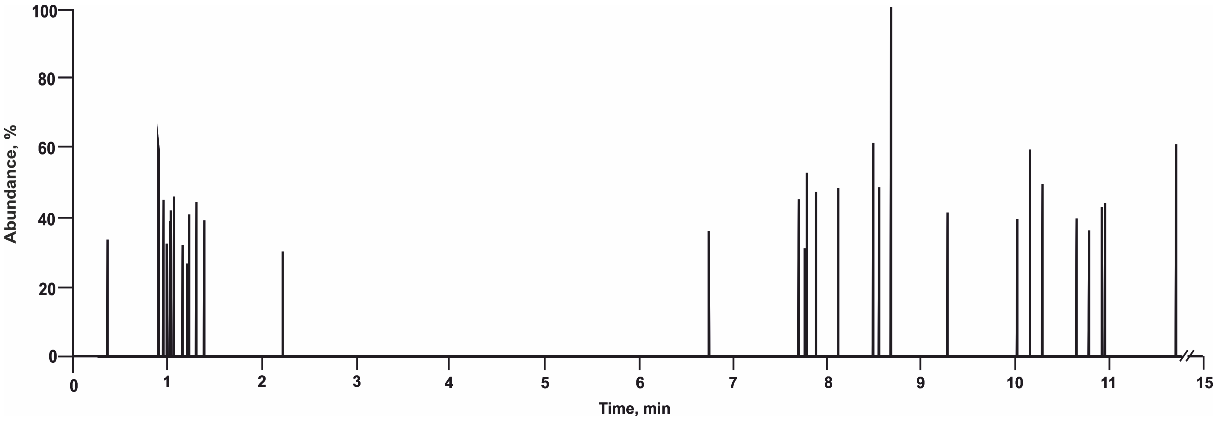


**Fig. S2** Reconstructed ion chromatograms obtained after analysis of an authentic urine sample used for the selectivity study

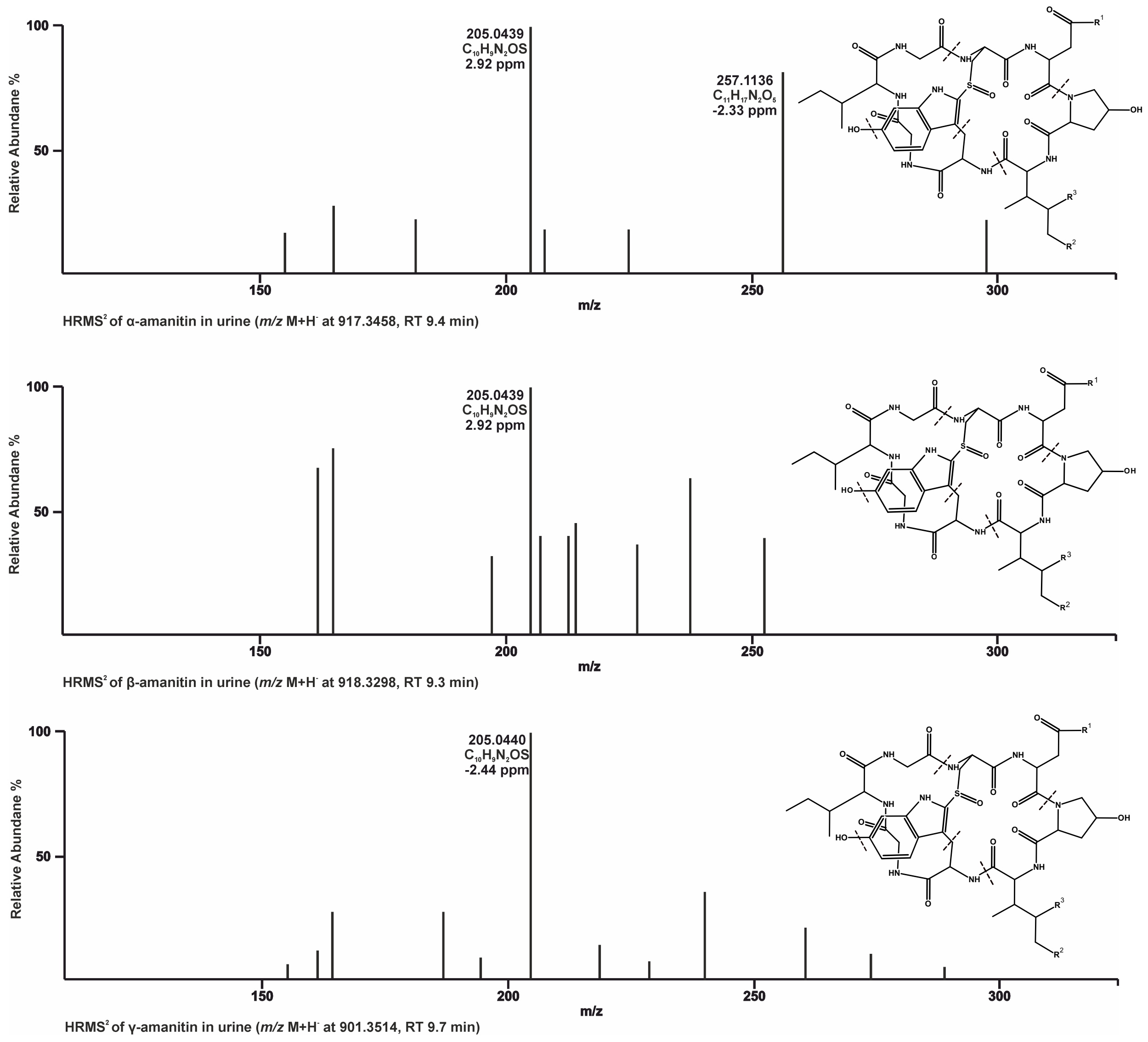


**Fig. S3** $HRMS^2$ spectra of the amatoxins, HRMS, high-resolution mass spectrometry, α-amanitin, $R_1 = NH_2$, $R_2 = OH$, $R_3 = OH$; β-amanitin, $R_1 = OH$, $R_2 = OH$, $R_3 = OH$; γ-amanitin, $R_1 = NH_2$, $R_2 = OH$, $R_3 = H$.
